# CRISPR/Cas9-Mediated Knockout of *HOS1* Reveals Its Role in the Regulation of Secondary Metabolism in *Arabidopsis thaliana*

**DOI:** 10.3390/plants10010104

**Published:** 2021-01-06

**Authors:** Yury Shkryl, Yulia Yugay, Tatiana Avramenko, Valeria Grigorchuk, Tatiana Gorpenchenko, Olga Grischenko, Victor Bulgakov

**Affiliations:** Federal Scientific Center of the East Asia Terrestrial Biodiversity, Far Eastern Branch, the Russian Academy of Sciences, 690022 Vladivostok, Russia; yuya1992@mail.ru (Y.Y.); avramenko.dvo@gmail.com (T.A.); kera1313@mail.ru (V.G.); gorp76@mail.ru (T.G.); crab_ol@mail.ru (O.G.); bulgakov@biosoil.ru (V.B.)

**Keywords:** Cas9, CRISPR, genome editing, *Arabidopsis thaliana*, glucosinolates, flavonoids, ICE1, HOS1, abiotic stress

## Abstract

In *Arabidopsis,* the RING finger-containing E3 ubiquitin ligase HIGH EXPRESSION OF OSMOTICALLY RESPONSIVE GENES 1 (HOS1) functions as a main regulator of the cold signaling. In this study, CRISPR/Cas9-mediated targeted mutagenesis of the *HOS1* gene in the first exon was performed. DNA sequencing showed that frameshift indels introduced by genome editing of *HOS1* resulted in the appearance of premature stop codons, disrupting the open reading frame. Obtained *hos1*^Cas9^ mutant plants were compared with the SALK T-DNA insertion mutant, line *hos1-3*, in terms of their tolerance to abiotic stresses, accumulation of secondary metabolites and expression levels of genes participating in these processes. Upon exposure to cold stress, enhanced tolerance and expression of cold-responsive genes were observed in both *hos1-3* and *hos1*^Cas9^ plants. The *hos1* mutation caused changes in the synthesis of phytoalexins in transformed cells. The content of glucosinolates (GSLs) was down-regulated by 1.5-times, while flavonol glycosides were up-regulated by 1.2 to 4.2 times in transgenic plants. The transcript abundance of the corresponding MYB and bHLH transcription factors, which are responsible for the regulation of secondary metabolism in *Arabidopsis,* were also altered. Our data suggest a relationship between HOS1-regulated downstream signaling and phytoalexin biosynthesis.

## 1. Introduction

Clustered regularly interspaced short palindromic repeats (CRISPR)/CRISPR-associated protein 9 (Cas9) technology has become a versatile approach of targeted genetic manipulations in plants [1,2]. In contrast to the classical loss-of-function approach introducing random mutations, Cas9 protein driven by an engineered 20-nt RNA sequence (short guide RNA; sgRNA) performs strand scission of a specific target sequence within the genome, enabling precise corrections [3]. Mutations introduced by Cas9/sgRNA are inherited in the germline and have a classical pattern of Mendelian segregation in the next generations, as has been demonstrated in the transformants of *Arabidopsis*, rice and tomato [4,5,6]. Amino acid substitutions in HNH or RuvC nuclease domains of Cas9 result in gaining nickase activity of Cas9 variants, while mutations introduced in both domains simultaneously result in catalytically dead Cas9 (dCas9), lacking its endonuclease activity [7]. Nevertheless, dCas9 is still capable of binding to its target sites of DNA [7]. Cas9 nickase and dCas9, coupled with other functional enzymes, may be used as carriers to a certain region in the genome targeted by sgRNA. Using this technique, gene activation or repression, single nucleotide substitutions and epigenetic editing of chromatin status have been reported [8].

Exposure to adverse environmental conditions, including extreme temperatures, water deprivation, excessive soil salinity, high radiation and chemical pollution, can negatively impact plant quality, accumulation of biomass and yield [9,10]. Different strategies in plant biotechnology have been developed to increase resistance and mitigate stress responses in plants, including overexpression of antioxidant genes [11,12], stress-responsive genes and transcription factors [13,14,15] or genes participating in biosynthesis of osmoprotectants [16,17]. Recently, CRISPR/Cas9-mediated mutagenesis of negative regulators of stress-responsive pathways became an alternative way of producing stress-tolerant plants [18,19]. The exploitation of the strategy could potentially help to overcome legal and social debates addressing the safety issues of genetically modified crops [20,21].

Transcriptional regulation of stress-responsive genes is one of the main defense mechanisms realized in plants exposed to cold [22,23]. C-repeat (CRT)-binding factors (CBFs) are able to bind to CRT or dehydration-responsive (DRE) *cis*-acting elements in the promoter region of the cold-responsive (COR) genes to activate their transcription [24,25]. When plants are exposed to low-temperature stress, *CBF* expression is rapidly induced, followed by subsequent upregulation of downstream genes responsible for cold adaptation [26]. Members of the MYC (basic helix-loop-helix, bHLH), WRKY, MYB and NAC transcription factor families are also known to be involved in cold stress response, emphasizing the importance of transcriptional control [23,27].

*A. thaliana* HOS1 is a RING finger E3 ubiquitin ligase [28]. HOS1 negatively regulates INDUCER OF CBF EXPRESSION 1 (ICE1), mediating its ubiquitination and proteolysis [28]. Degradation of ICE1 protein is stimulated under cold treatment, whereas its disruption is inhibited in loss-of-function *hos1* mutants. Moreover, plants constitutively expressing *HOS1* showed reduced freezing tolerance accompanied by decreased level of *CBF* transcripts [28]. The HOS1 participates in the regulation of flowering time by interacting with phyB to promote the degradation of CONSTANS (CO) via 26S proteasome pathway [29]. CO activates transcription of the floral integrators, *FLOWERING LOCUS T* (*FT*) and *TWIN SISTER OF FT* (*TSF*) genes, which in turn govern the flowering time of *A. thaliana* [30]. Moreover, HOS1 protein is involved in maintenance of the chromatin structure independent of its primary activity. Expression of the floral repressor *FLOWERING LOCUS C* (*FLC*) is activated via HOS1-mediated chromatin remodeling to control flowering under short-term cold treatment [28,31]. Ectopic expression of the *HOS1* gene provokes a two-fold increase of *FLC* mRNA abundance [31]. Additionally, HOS1 affects AGO1 homeostasis by controlling the accumulation of *miR168a/b* targeting the *ARGONAUTE 1* (*AGO1*) gene transcript in *Arabidopsis* [32].

To date, 16 T-DNA insertional mutant lines for *HOS1* gene of *Arabidopsis* have been deposited in the Salk Institute collection. Annotated T-DNA insertion positions include exons (from the third to eighth) and intronic regions of the *HOS1* gene [31,32,33,34,35,36,37,38]. Detailed characterization of the obtained lines revealed *hos1* mutants to be highly pleiotropic, pointing out the unequal effects of the mutations [31,34]. Among deposited *hos1-3* mutant has been intensively investigated and characterized best [29,32,34,35,37]. In this work, using the CRISPR/Cas9 chemistry, we introduced mutations in the first exon of the *HOS1* gene and established *hos1*^Cas9^ mutant lines of *A.* *thaliana*. Abiotic stress tolerance, accumulation of secondary metabolites and expression of the biosynthetic genes were investigated in obtained mutants and SALK *hos1-3* lines. Our results show that *HOS1* is necessary for the glucosinolate biosynthesis, because both Cas9-engineered and T-DNA insertion mutants showed significant reductions in their content. In contrast, HOS1 appears to be a negative regulator of flavonoid biosynthesis, since *hos1* plants exhibit a strong upregulation of flavonol glycosides.

## 2. Materials and Methods

### 2.1. Plant Material and Growth Conditions

Wild-type *Arabidopsis thaliana* (Columbia-0) seeds were kindly provided by Dr. Jen Sheen (Harvard Medical School, Boston, MA, USA). Seeds were subjected to cold stratification for 2 days at 4 °C in darkness, followed by transfer to a half-strength of W medium [39], containing sucrose (10 g/L) and agar (8 g/L). The seeds were germinated at 25 °C under 16 h light/8 h dark photoperiod (long-day conditions, LD) in a growth chamber. Ten-day-old seedlings were transferred to soil for further growth.

*A*. *thaliana* SALK_069312C T-DNA insertion line was purchased from the Salk Institute for Biological Studies, San Diego, CA, USA. This line has been previously termed *hos1-3* [34] and carries a single T-DNA copy in the fifth exon of the *HOS1* gene. Seeds of the *hos1-3* mutants were germinated as described above.

### 2.2. sgRNA Design and Plasmid Construction

**Figure 1:**
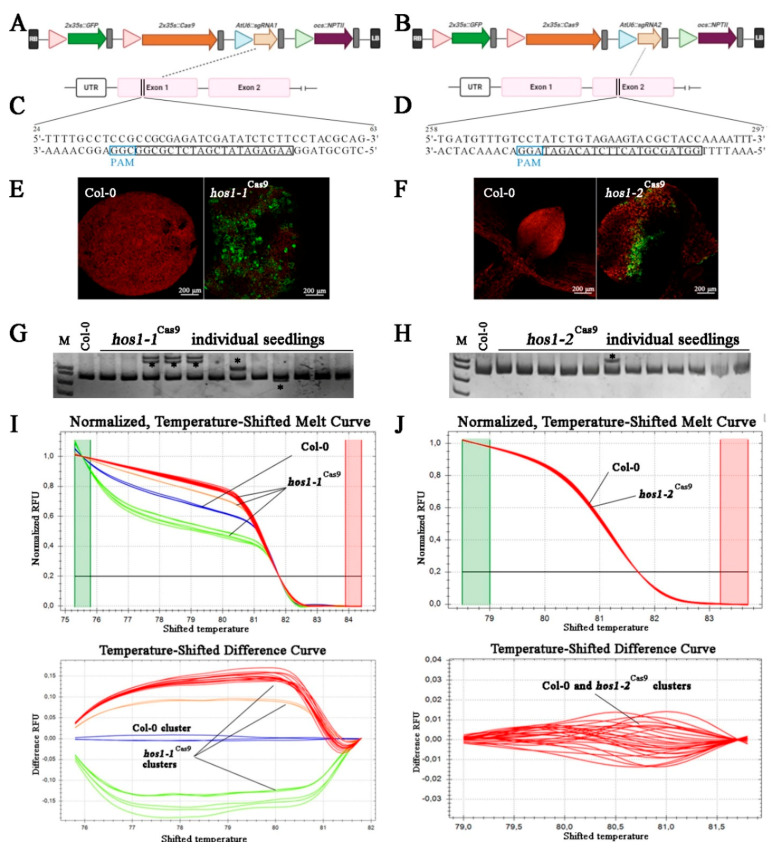
Schematic illustration of the CRISPR/Cas9 vectors, gRNA target sites, GFP fluorescence in transiently transformed seedlings and mutant screening. (**A**,**B**) The physical map of T-DNA fragment of binary vectors used in this study. (**C**,**D**) The target sequences for mutagenesis with gRNA1 and gRNA2. The selected protospacer sequences are shown in black boxes and the protospacer adjacent motifs (PAMs) are in blue boxes. (**E**,**F**) Visualization of green fluorescent protein (GFP) fluorescence in *A. thaliana* seedlings subjected to *Agrobacterium*-mediated transformation using vacuum infiltration. (**G**,**H**) Heteroduplex mobility assay (HMA) of *A. thaliana* seedlings transformed with the CRISPR/Cas9 using polyacrylamide gel electrophoresis (PAGE). Asterisks (*) indicate heteroduplex bands. (**I**,**J**) High resolution melting (HRM) analysis of amplicons corresponding to targeted *HOS1* gene regions in transformed *A. thaliana* seedlings.

Plasmids HBT-pcoCas9 and pUC119-gRNA (Figure 1A) containing CRISPR/Cas9 elements [40] were purchased from Addgene (https://www.addgene.org/). Plant codon-optimized Cas9 variant form HBT-pcoCas9 was inserted as a *Nco*I-*Pst*I fragment into pSAT4-MCS expression cassette under the regulation of the double CaMV 35S promoter [41]. Constructed 2 × 35S-pcoCas9 expression cassette was then excised as an I-*Sce*I fragment and ligated into the binary vectors pPZP-RCS2-NPTII and pPZP-RCS2-NPTII/EGFP [41].

Two sgRNAs spacers targeting the first (gRNA1; CCGCGAGATCGATATCTCTT) and second (gRNA2; ATCTGTAGAAGTACGCTACC) exons of *A. thaliana HOS1* gene (GenBank accession no. NM_129540) were designed using an online tool (ATUM). Site-directed mutagenesis using gRNA-specific primers (Table S1) and pUC119-gRNA were employed to introduce the designed protospacer sequences in a frame with gRNA scaffold under the control of the AtU6 promoter. In order to pre-validate the functionality of the designed sgRNAs, we performed in vitro cleavage analysis of target sites with synthetic sgRNA obtained using HiScribe T7 Quick High-Yield RNA Synthesis Kit (NEB, Ipswich, MA, USA) and Cas9 nuclease (NEB), which confirmed the efficiency of Cas9 cleavage with sgRNAs designed for *HOS1* (Figure S1).

By using polymerase chain reaction (PCR) with a primer pair, containing I-*Ceu*I restriction sites (Table S1), the resulting sgRNA expression cassettes were directly ligated into the same sites of the linearized binary vectors. The final constructs pPZP-RCS2-NPTII/pcoCas9/gRNA1, pPZP-RCS2-NPTII/pcoCas9/gRNA2, pPZP-RCS2-NPTII/pcoCas9/gRNA1/EGFP and pPZP-RCS2-NPTII/pcoCas9/gRNA2/EGFP, were electroporated into *Agrobacterium tumefaciens* strain EHA105/pTiBo542 [42] by using Gene Pulser (Bio-Rad Laboratories, Inc., Hercules, CA, USA) according to the manufacturer’s protocol. Cells were grown overnight on Luria-Bertani (LB) agar plates containing 300 mg/L spectinomycin, 200 mg/L streptomycin and 150 mg/L rifampicin, at 28 °C and verified by PCR with corresponding gene-specific primers.

### 2.3. Transient Transformation of A. thaliana Seedlings

Transient transformation of *Arabidopsis* seedlings was performed by vacuum infiltration, in accordance with the protocol described by Reference [43]. The day before infiltration, *A. tumefaciens* harboring pPZP-RCS2-NPTII/pcoCas9/gRNA1/EGFP or pPZP-RCS2-NPTII/pcoCas9/gRNA2/EGFP constructions were picked from the plate, inoculated into 20 mL of LB medium with antibiotics and cultivated overnight at 28 °C with moderate shaking (150 rpm). The optical density (OD_600_) of *Agrobacterium* suspension was measured using BioSpec-nano spectrophotometer (Shimadzu Corporation, Kyoto, Japan). Then overnight liquid culture was centrifuged at 4000× *g* for 10 min at room temperature (24 °C), the pellet was then resuspended in the appropriate volume of infiltration buffer (10 mM MES, pH 5.6, 10 mM MgCl_2_, 100 µM acetosyringone) to give a final OD_600_ of 0.3. Three-four-day seedlings were immersed into a bacterial suspension placed in a 50-mL Falcon sterile tube and a short-term vacuum treatment was applied twice. Immediately after infiltration, the seedlings were washed three times with sterile water. Afterward, plants were transferred to half-strength W medium without antibiotics for cocultivation. After 3 days of cocultivation, the seedlings were washed in sterile distilled water and used for confocal GFP fluorescence analysis and DNA isolation.

### 2.4. Laser Confocal Imaging of EGFP in Living Cells

The detection of the GFP fluorescence in transformed seedlings was performed using the LSM 710 LIVE and LSM 510 META scanning confocal laser microscopes (Carl Zeiss, Jena, Germany) with an excitation wavelength of 488 nm and an emission filter of 505–530 nm. Non-transformed seedlings were used as negative control. To monitor emission maximum of GFP, spectral imaging in the 505–720 nm range with a 10.4 nm detection bandwidth was performed in all examined transformed plants. Wavelength scanning was employed to check the specificity of the GFP signal. Video files and single frames of the captured images were recorded and analyzed with LSM 510 Release version 4.2 and ZEN 2011 software. The absence of autofluorescence was evaluated using the λ-scan mode (Figure S2).

### 2.5. Obtaining of Stable hos1^Cas9^ Mutant Plants

The pPZP-RCS2-nptII/pcoCas9/gRNA1 and pPZP-RCS2-nptII/pcoCas9/gRNA2 constructs were transformed into *A*. *thaliana* by the floral dip method [44]. T1 plants were selected in Petri dishes on a half-strength of W medium containing 50 µg/L kanamycin. Antibiotic-resistant transformants were further transplanted on antibiotic-free medium in glass tubes for continuous growth, molecular analysis and seed harvesting. Homozygous T3 transgenic plants were used in this study. Plant growth conditions for transgenic plants were the same as for wild-type (WT) counterparts.

### 2.6. Genotyping and Sequencing of the HOS1 Gene Mutations

DNA extraction from leaves was performed using the cetyl trimethylammonium bromide (CTAB) protocol (Molecular Cloning, 3rd edition). Gene-specific primer pairs *HOS1-1*-target and *HOS1-2*-target (Supplementary Table S1) were used to amplify 381- and 372-bp long genomic regions containing the corresponding target sites, respectively. Obtained amplicons were ligated into a pJET (Thermo Fisher Scientific Inc., MA, USA) and sequenced using an ABI 3500 Genetic Analyzer (Applied Biosystems, Foster City, CA, USA).

To genotype mutations by a heteroduplex mobility assay (HMA), polyacrylamide gel electrophoresis (PAGE) analysis of amplicons subjected to denaturation/renaturation cycle was performed following the procedure of Reference [45]. To screen for mutations using high-resolution melting (HRM) analysis, we performed polymerase chain reaction (PCR)-HRM reactions with the same primer sets and 2.5 x SYBR green PCR master mix (Evrogen, Moscow, Russia) using CFX96 thermocycler (Bio-Rad Laboratories). Melting curves were determined by incubating the reaction mixes from 60 °C to 95 °C with an increment 0.2 °C for 10 s with a plate reading. Precision Melt Analysis software (Bio-Rad Laboratories) was utilized to discriminate between native and mutant *HOS1* alleles.

### 2.7. Gene Expression Analysis

Total RNA from leaves of *A. thaliana* plants was isolated using LiCl protocol [46]. The technique of RNA analysis and first strand cDNA synthesis was described by us previously [47]. Quantitative PCR (qPCR) analysis was conducted using CFX96 thermocycler (Bio-Rad Laboratories) with 2.5 x SYBR green PCR mix (Evrogen) as described [48]. The primer sets used in the qPCR are listed in Table S1. Analysis for three biological replicates from three separate RNA extractions and three technical replicates for each qPCR experiment were performed. CFX Manager Software (Version 3.1; Bio-Rad Laboratories) was used for data processing. Melting curve analysis was conducted after each run to verify the absence of primer-dimer artefacts or non-specific amplicons. An expression-based heat map was created using web server Heatmapper [49].

Five candidate reference genes including ubiquitin (*UBQ,* GenBank accession no. AT4G05320), glyceraldehyde-3-phosphate dehydrogenase (*GAPDH*, GenBank accession no. AT1G13440), elongation factor-1 alpha (*EF-1α*, GenBank accession no. AT5G60390), TAP42-interacting protein of 41 kDa (*TIP41*, GenBank accession no. AT4G34270) and a SAND family protein monensin sensitivity 1 (*MON1*, GenBank accession no. AT2G28390) were validated for their expression stability (Figure S3). The relative fold change in mRNA abundance was calculated using the RefFinder [50], which integrates several algorithms (BestKeeper, Normfinder, comparative deltaCt method and the geNorm) to reveal the best reference genes. Most stable genes *MON1* and *TIP41* were used in this study as internal controls to normalize gene expression levels.

### 2.8. HPLC-MS Analysis

Identification of secondary metabolites in dry aerial parts of 6-week-old control and transgenic plants was performed on a 1260 Infinity analytical high-performance liquid chromatography (HPLC) instrument (Agilent Technologies, Santa Clara, CA, USA) coupled with an electrospray ionization (ESI) ion trap mass spectrometer (MS) (Bruker HCT ultra PTM Discovery System, Bruker Daltonik GmbH, Bremen, Germany). Samples were separated into fractions on a Zorbax C18 column (Agilent Technologies). Kaempferol, quercetin, sinigrin hydrate and sulfatase (H-1 from Helix pomatia) standards were obtained from Sigma-Aldrich (St. Louis, MO, USA). Experimental procedure of sample preparation and glucosinolate desulfation, as well as chromatographic and mass spectrometric parameters, has been previously described in detail [48]. Quantitative analysis was performed with a photodiode array detector using commercially available reference standards. Agilent OpenLAB CDS software (v.01.06.111) was used for data processing. Representative MS spectra (acquired in negative ionization mode) were obtained for each sample to confirm target profiling of metabolites. The Bruker Daltonics Compass 1.3 esquire control software (v.6.2.581.3 and v.4.0.234.0) was used for collecting and processing MS data.

### 2.9. Statistical Analysis

Statistical tests were performed using Statistica 10.0 (StatSoft Inc., Tulsa, OK, USA) with the threshold for statistical significance set at *p* < 0.05. PAST 4.03 software was used for principal component analysis (PCA) of secondary metabolites detected by HPLC-MS [51]. Two independent categories were compared using the Student’s t-test, while ANOVA analysis followed by a multiple comparison method was applied when comparisons among multiple groups have been performed. The inter-group comparison was made using Fisher’s protected least significant difference (PLSD) post-hoc test.

## 3. Results and Discussion

### 3.1. Assessment of sgRNA Activity Using Transient Transformation

We constructed plant CRISPR/Cas9 all-in-one binary vectors harboring components for *HOS1* mutagenesis, including the plant codon-optimized Cas9 (pcoCas9) and enhanced green fluorescent protein (EGFP), both controlled by the 35S CaMV promoter, a sgRNA expression cassette driven by the AtU6-1 promoter and the *NPTII* gene as a negative selectable marker (Figure 1A,B). In these vectors, Cas9 contains nuclear localization signals (NLSs) at N- and C-termini, which ensure efficient nuclear targeting [40].

*A. thaliana HOS1* gene (GenBank No. NM_129540) is located in chromosome 2 and is 5346 bp in size, with 10 exons. Two gRNAs targeting exon 1 and 2 of the *HOS1* gene were designed (Figure 1C,D), CRISPR-OFFinder was used for off-target scoring [52]; no potential off-targets were detected with 0 to 2 mismatches in the core sequence.

Pre-evaluation of genetic constructions in a particular plant is an important step for validation of genome engineering experiment. Although computational tools are widely used to predict gRNA efficiency based on the sequence analysis, the calculated on-target activity often does not match the results obtained *in planta* [53,54]. To test the effectiveness of CRISPR/Cas9, a system of transient expression of genetic constructs in protoplasts or plant seedlings is utilized for the screening of DNA mutations [55].

We performed *Agrobacterium*-mediated transient transformation of *A. thaliana* seedlings by vacuum infiltration method. Successful transformants were selected by detecting GFP fluorescence in leaves (Figure 1E,F), which could potentially indicate that Cas9 and sgRNA cassettes were also expressed at the appropriate levels. To determine the mutation efficacy of two selected *HOS1* gRNAs, we further conducted a heteroduplex mobility assay (HMA) using PAGE separation of heteroduplexes between the native allele of the gene and its mutant variant [45]. A total of 25 GFP-positive seedlings were screened for each of the gRNA construction and the typical PAGE results are depicted in Figure 1G,H. Heteroduplex bands were detected in PCR products of the gRNA1- and gRNA2-transformed seedlings, whereas a single band was observed in the wild-type sample, indicating that the CRISPR/Cas9-mediated mutations were introduced at the target site of the *HOS1* gene. According to the number of mutated bands, the efficiency for gRNA1 and gRNA2 in *Arabidopsis* was about 36% (9 mutant seedling) and 4% (1 mutant seedling), respectively. To verify the presence of mutant alleles of the *HOS1* gene in transformed seedlings, genotyping by the high-resolution melting (HRM) analysis of the PCR products was also performed [56]. Figure 1I,J shows an example of such an analysis, according to which the PCR products of mutant plant lines (denoted with red, orange and green curves) differed from the PCR products of control plants (blue curves). These results suggested that the obtained vectors were suitable to be used for plant transformation. However, gRNA1 showed almost 10-fold higher effectiveness of genomic editing than gRNA2. Different levels of sgRNA activity is a common phenomenon observed during CRISPR/Cas9-mediated genome editing. For example, in wheat protoplasts treated with seven *EPSPS*-specific sgRNAs, the mean frequency of indels varied from 0.0 to 23.3% [57]. An intensive screening of 195 gRNAs in transiently transformed tomato leaves revealed that 49% of tested gRNAs showed 0–1% mutation efficiency [58]. It has been previously demonstrated that sgRNAs have similar editing efficiency in the transient assay and in stable transgenic plants [53].

### 3.2. Identification and Analysis of Stable Arabidopsis Mutants

**Figure 2:**
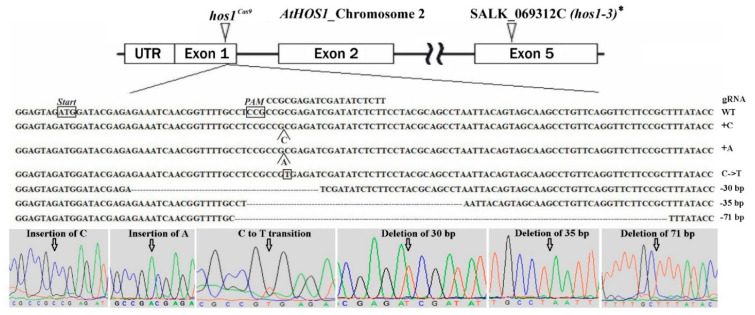
Schematic illustration of the gRNA1 target site in *HOS1* gene. The detail underneath shows partial sequences for the native and mutated *HOS1* alleles. The sequence chromatograms are taken from Trev 1.8b1 (Staden Package). The positions of each of the identified mutations are marked with arrows. * The precise position of T-DNA integration site within the *HOS1* gene and predicted sequence of the truncated protein in the *hos1-3* mutant line are shown in Figure S4 of Supplementary Materials.

*Agrobacterium*-mediated transformation of flower buds was performed to obtain *A. thaliana* transgenic plants [44]. Only the gRNA1 construct was used for transformation, since the gRNA2 activity was very low in the transient assay. T1 transformed plants were selected on media containing 50 μg/L kanamycin. Antibiotic-resistant seedlings were analyzed for the presence of the mutant *HOS1* allele using automated Sanger sequencing of cloned target PCR fragments. 246 clones were sequenced from 12 T1 transgenic plants and six types of mutations were revealed: two insertions, three deletions and one transition (Figure 2).

We should note that not all detected mutational events led to the inactivation of the HOS1 at the protein level (Table 1). In particular, the transition led to the replacement of alanine (A) with valine (V) and a deletion of 30 bp leads to the elimination of 10 aa without shifting the reading frame. Single nucleotide insertions and deletions of 35 and 71 bp resulted in a frameshift mutation producing premature termination codon. Only two homozygous mutants, *hos1-1*^Cas9^ and *hos1-2*^Cas9^, were revealed by genotyping and both of them carried 1-bp insertion (Table 1).

### 3.3. Plant Phenotype and Expression Profiles of Flowering Control Related Genes

**Figure 3:**
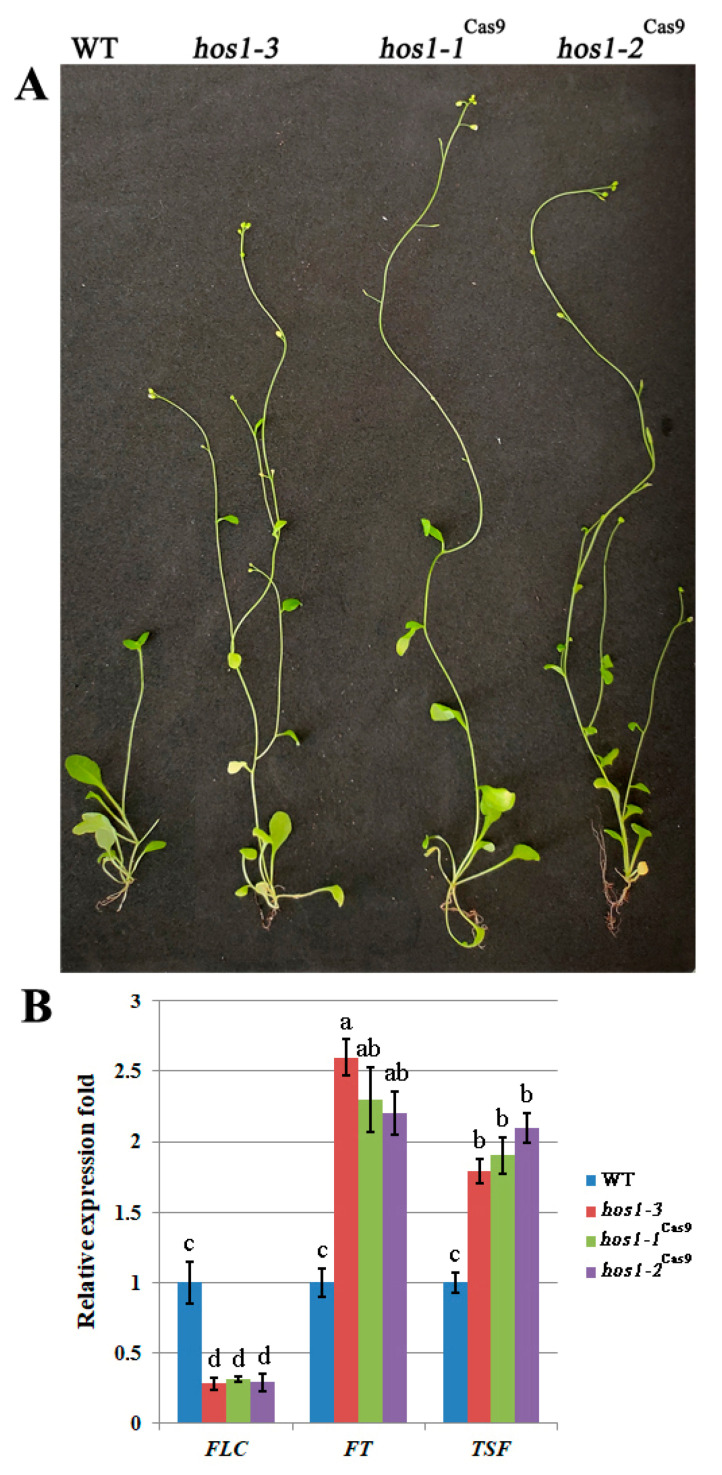
Phenotypic characterization of the *hos1*^Cas9^ mutants and expression of flowering-time genes. (**A**) Flowering phenotype of *hos1* mutant plants (*hos1-3*, *hos1-1*^Cas9^ and *hos1-2*^Cas9^) in comparison with wild-type (WT) genotype grown in LD conditions for 4 weeks; (**B**) qPCR analysis of *FLC*, *FT* and *TSF* expression. Data are presented as mean ± standard error. Different letters above the bars indicate statistically significant differences of means (*P* < 0.05) for each of the genes (Fisher’s LSD).

The HOS1 protein negatively regulates the CO protein levels thus modulating flowering time [34]. Consistent with previous results [35], the SALK *hos1-3* plants initiated flowering at leaf number 9, while wild-type plants it was at number 14. Like previously established *hos1* mutant alleles, the newly isolated *hos1-1*^Cas9^ and *hos1-2*^Cas9^ mutant alleles displayed early flowering at the same number of leaves as *hos1-3* mutants (Figure 3A). This result indicates that of *hos1*^Cas9^ mutation did not provoke additional effect on the phenotype of transgenic plants. In accordance with previous observations [34], *FLC* transcriptional level was considerably decreased in *hos1-3* plants (Figure 3B). On the contrary, the transcription of flowering integrator genes, *FT* and *TSF*, was up-regulated in the *hos1-3* line. Our *hos1-1*^Cas9^ and *hos1-2*^Cas9^ mutant plants showed similar patterns of *FLC*, *FT* and *TSF* expression (Figure 3B). Activation of *FT* was slightly less effective in *hos1-1*^Cas9^ and *hos1-2*^Cas9^ plants, compared with the *hos1-3* line. *TSF* was activated slightly stronger in the *hos1-2*^Cas9^ plants, compared to *hos1-1*^Cas9^ and *hos1-3* mutants. It has been previously proposed that HOS1 could potentially mediate the crosstalk between photoperiod and temperature regulatory factors of flowering, thus facilitating plant adaptation to environmental signals [34,35].

### 3.4. Abiotic Stress Treatment and Expression of CBF Regulon Genes

**Figure 4:**
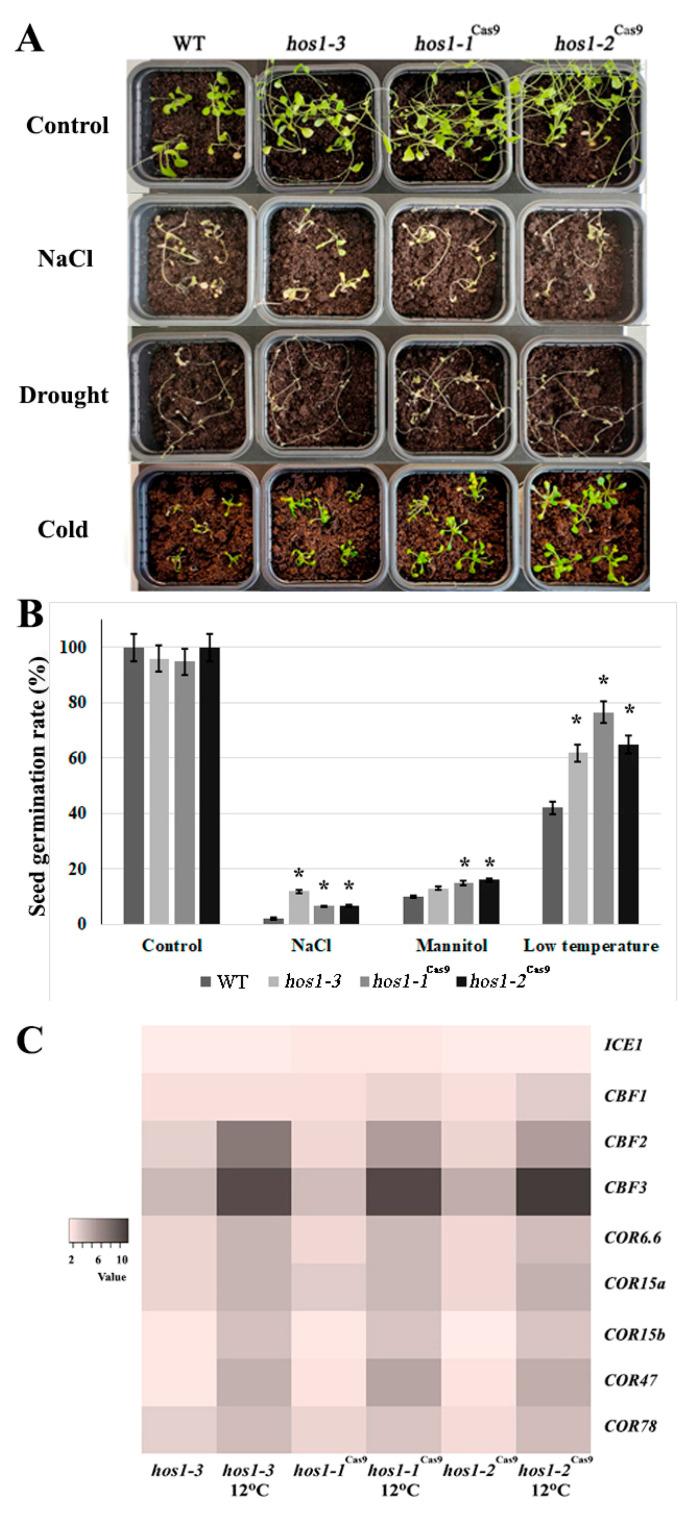
Abiotic stress treatment and expression of CBF regulon genes. (**A**) Phenotypic comparison of the plants under salt, drought and cold stresses. (**B**) Seed germination rates of *hos1-3* and *hos1*^Cas9^ plants under abiotic stress treatment. Data are presented as mean ± standard error. Asterisks (*) indicate statistically significant differences of means (*P* < 0.05), Student’s t-test. (**C**) A heatmap of the expression fold changes of *ICE1*, *CBF and COR* genes in *Arabidopsis hos1* mutants, under normal growth conditions and under cold stress treatment, relative to their expression levels in WT plants.

*Hos1* mutants exhibited transcriptional activation of cold-responsive genes and higher tolerance to cold stress [28]. In order to evaluate if Cas9-mediated *HOS1* mutations affect the cold signaling, we subjected *hos1-3*, *hos1-1*^Cas9^ and *hos1-2*^Cas9^ mutants to continuous cold stress at 12 °C exposure for 2 weeks (Figure 4A). All *hos1* mutants showed similar flowering time under normal and cold conditions (at leaf number 12), while the flowering time of Col-0 plants was considerably delayed (at leaf number 23). There was no visible difference in *hos1* plants grown in high NaCl concentration and without watering compared with control plants (Figure 4A). However, the seed germination test showed that *hos1-3* and *hos1*^Cas9^ plants had higher seed germination rates under cold stress treatment (12 °C) on medium containing NaCl (300 mM) and mannitol (300 mM), compared to the corresponding control conditions (Figure 4B).

The CBF/DREB1 regulon, which controls the transcription of hundreds of genes activated by cold, is known to be one of the major stress-signaling pathways affecting cold tolerance [59]. The CBF/DREB1 genes are controlled partially by ICE1, which was considered to be the main regulator during cold acclimation in *A. thaliana* [60]. Transgenic plants with increased expression of *ICE1* and *CBF* genes showed considerable resistance to cold temperatures [61].

We studied the expression of key components of the CBF/DREB1 regulon in *hos1-3*, *hos1-1*^Cas9^ and *hos1-2*^Cas9^ mutants subjected to continuous 12 °C exposure for 2 weeks (Figure 4C). The expression of *ICE1* in the *hos1* mutant plants was almost equal to control. The *hos1* mutation caused significant activation of the *CBF2* and *CBF3* genes, while the level of *CBF1* expression was activated to a lesser extent. As previously reported, no alterations in *HOS1* transcript abundance was detected at ambient temperatures and the expression of *CBF* genes was not considerably affected by altered HOS1 activity without cold treatment [28].

When exposed to cold stress, *hos1-3* and *hos1-1*^Cas9^ plants exhibited strong transcriptional activation of the cold-responsive genes, with the exception of *ICE1* (Figure 4C). Among the *CBF* genes, *CBF2* and *CBF3* were up-regulated in *hos1* mutant lines up to 8–12 times compared with the control plant. The transcriptional abundance of *COR* genes was also considerably higher in both *hos1-3* and *hos1*^Cas9^ plants: their expression was activated by 4 to 5-fold.

COR proteins play a major role in physiological rearrangements, leading to an increase of cold resistance. About 4000 COR genes are known in *Arabidopsis* [62], while factors CBF1, CBF2 and CBF3 regulate only about 10% of them [63]. Of the CBF-regulated *COR* genes of *Arabidopsis*, the most studied are *COR6.6*, *COR15a*, *COR15b*, *COR47* and *COR78* [64]. We examined the expression of these genes in Cas9-edited and SALK mutant *Arabidopsis* plants (Figure 4C). The levels of transcription of most of the *COR* genes in the studied lines were up-regulated compared to control. Expression of such genes as the *COR6.6, COR15a* and *COR78* in *hos1* mutant plants exceeded that of the control plant by almost 3-fold.

### 3.5. Secondary Metabolites Content and Expression of the bHLH and MYB Transcription Factors in hos1-3 and hos1^Cas9^ Mutants

HPLC analysis revealed that the *hos1* mutant *Arabidopsis* plants showed remarkably altered secondary metabolite profiles (Table 2). We found that the levels of total flavonoids were up-regulated by 1.2 to 4.2 fold in *hos1* plants and this effect was mainly due to the rise of kaempferol derivatives content. Principal component analysis based on the content of the flavonol glycosides identified two significant components; PC 1 explained 97.4% of the variance, while PC 2 had a variation of 1.9%. The loading plot of PC 1 was dominated by kaempferol hexose dideoxyhexose, kaempferol hexose deoxyhexose and kaempferol dideoxyhexose. PC 2 was mainly contributed by kaempferol hexose deoxyhexose. The content of glucosinolates (GSLs) in the mutant lines was reduced up to 1.5 times (Table 2). At the same time, clear patterns of change in certain metabolites of this group in *hos1* mutant lines were not obvious. The strongest decrease was noted for aliphatic GSLs (methyl-thioalkyl and methyl-sulfinylalkyl GSLs), however, the reason for this selective action is not clear. Principal component analysis based on the content of glucosinolates identified three significant components; PC 1 had a variation of 52.1%, while PC 2 explained 40.4% of the variance and PC 3 had 7.5%. The loading plot of PC 1 was mainly contributed by 3-methylsulfinylpropyl glucosinolate, 4-methylsulfinylbutyl glucosinolate and 5-methylsulfinylpentyl glucosinolate. PC 2 was dominated by 4-methylthiobutyl glucosinolate, 8-methylthiooctyl glucosinolate and 8-methylsulfinyloctyl glucosinolate.

**Figure 5:**
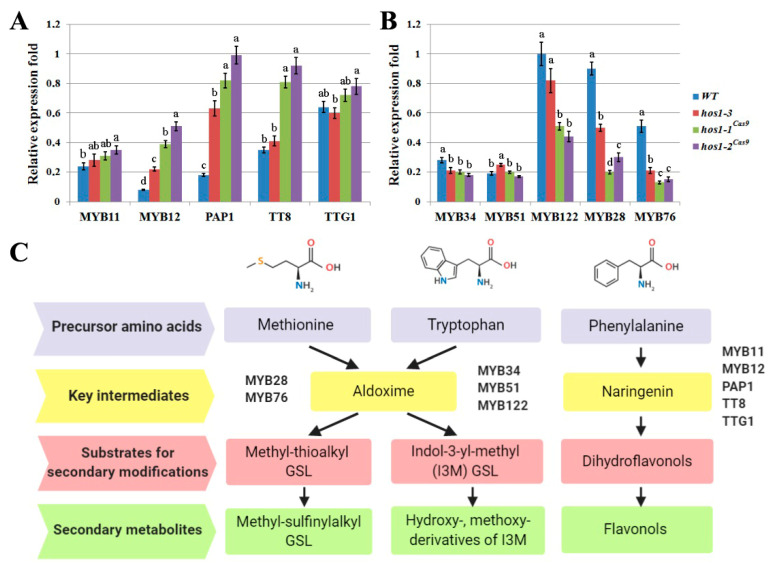
The relative differential expression of transcription factors involved in the biosynthesis of secondary metabolites in *A*. *thaliana*. (**A**) The expression of *MYB* and *bHLH* genes family members implicated in biosynthesis of flavonoids. (**B**) The expression of MYB genes implicated in biosynthesis of glucosinolates (GSLs). Data are presented as mean ± standard error. Different letters above the bars indicate statistically significant differences of means (*P* < 0.05) for each of the genes (Fisher’s LSD). (**C**) Schematic representation of biosynthetic pathways of the particular groups of *A. thaliana* secondary metabolites and its transcriptional activators.

We next examined the level of expression of the bHLH and MYB transcription factors that regulate secondary metabolism in *Arabidopsis* (Figure 5C). The expression of the gene encoding the flavonoid-specific transcription factor MYB12 was increased by 3 to 6 times the *hos1-3* and *hos1*^Cas9^ mutant lines, compared with the control line (Figure 5A). Expression of *MYB11* in *hos1-3* and *hos1*^Cas9^ mutant lines did not differ from the control plants. It has been reported that enhanced expression of *MYB12* in *Arabidopsis* led to increase in flavonoids production [65]. Probably, activation of flavonoid biosynthesis in *hos1* mutants was caused by the up-regulation of *MYB12*. The expression analysis of the MYB-bHLH-WD40 components showed that TTG1, an important regulator of late structural genes in biosynthesis of flavonoids [66], was expressed in a similar manner in WT, *hos1-3 and hos1*^Cas9^ plants (Figure 5A). Expression of the transcription factor genes *TT8* and *PAP1* (synonym: *MYB75*) was strongly activated in *hos1-3* and *hos1^Cas9^* mutants. It was shown that *TT2* and *PAP1* overexpression is sufficient for late flavonoid gene activation in *A. thaliana* protoplasts with comparatively low levels of *TTG1* [67,68]. Similar results were obtained in *A. thaliana* seedlings with knockout of the *MYBL2* gene, which encodes an R3-MYB-related protein. Two independent *mybl2* mutants showed enhanced accumulation of anthocyanins and this effect was accompanied by the increase in transcriptional activity of *TT8* and *PAP1* genes, while *TTG1* was not affected [69]. Therefore, our results indicate that transcriptional activity of *MYB12, TT8 and PAP1* in *hos1* mutants was sufficient to mediate overproduction of flavonoids.

The expression of aliphatic GSLs related genes, *MYB28* and *MYB76*, was found to be considerably decreased in *hos1* mutants compared to the control (Figure 5B). MYB28 and MYB29, together with MYB76, are the main regulators of aliphatic GSLs biosynthesis in *Arabidopsis*. Among these genes, *MYB28* and *MYB29* are equally important for activation of GSLs biosynthesis genes, because *myb28myb29* double mutation completely abolished accumulation of aliphatic GSLs and the expression of *MYB76* alone did not neutralize the loss of their function [70]. However, genes involved in the production of indolic GSLs responded differently. In the *hos1-3* and *hos1*^Cas9^ mutants, expression of *MYB34* and *MYB122* decreased, whereas the expression of *MYB51* was generally stable (Figure 5B). It is known that MYB34 and MYB51 play pivotal roles in the biosynthesis of indolic GSLs in *A. thaliana*, while MYB122 has an accessory role and its function becomes evident during specific environmental stimuli [71]. Thus, the expression pattern of TFs regulating the biosynthetic pathways of aliphatic and indolic GSLs in the *hos1* mutant plants is consistent with the content of secondary metabolites in them.

Although the effect of *hos1-3* and *hos1^Cas9^* mutations on flowering time and abiotic stress responses was equal, secondary metabolism was modified differently in these lines. In particular, accumulation of flavonol glycosides was mainly increased in *hos1^Cas9^* mutants, while inhibition of indolic glucosinolates level was less pronounced in *hos1-3* plants (Table 2). The *hos1-3* mutation is located in the 5th exon and disrupts *HOS1* reading frame at position 912 bp of mRNA leading to the production of a truncated protein which, however, retains 304 aa of a native form (Figure S4). This protein lacks an important C-terminal portion of the polypeptide including protein-protein interaction domain and a nuclear localization signal but still contains the intact RING finger E3 ligase domain located at aa 34–100. It has been previously noted that different *hos1* variants exhibit pleiotropic effects on many processes of plant physiology [72]. Moreover, in vitro ubiquitination assay revealed that activity of a truncated HOS1 having 1–550 aa was similar to native protein, while another mutant form having only 1–210 aa was not able to generate ubiquitinated products [28]. Currently there is no information about potential ubiquitin ligase activity of a 1–304 aa truncated HOS1 in *hos1-3 Arabidopsis* mutant line. However we cannot fully exclude that it may possess some nonspecific function thus conferring changes in biosynthesis of secondary metabolites different from those of *hos1^Cas9^* lines.

The molecular mechanism by which loss of *HOS1* function mutation cause transcriptional perturbations of the bHLH and MYB TFs and subsequent secondary metabolite perturbations in *hos1-3* and *hos1*^Cas9^ mutants is not clear yet. HOS1 has a pivotal role in light and cold signaling and thus, its effect on phytoalexin biosynthesis may be attributed to these two main functions. Recently FLC was found to mediate the integration between flowering time regulation and biosynthesis of GSLs in *Arabidopsis* [73]. More specifically, *FLC* knockout mutation led to increased transcript levels of *MYB15*, while *MYB29* was down-regulated. The presence of sulfate transporter *SULTR2*;1 and *FLC* loci in two *Aethionema arabicum* ecotypes (CYP and TUR) is responsible for up to 10-fold difference in GSL concentrations, with the earlier-flowering CYP genotype always have the higher aliphatic GSLs concentration [74]. In addition, direct regulation of *Arabidopsis* indolic GSL biosynthetic gene, *CYP79B3*, is also possible because FLC binds to its promoter region [75].

Another possible mechanism can be realized in *hos1* mutants via participation of HOS1 in the modulation of CBF regulon function. In *Arabidopsis,* HOS1 triggers ubiquitination ICE1 [28] and this degradation is inhibited by *hos1-3* and *hos1^Cas9^* mutation. Cold treatment facilitates the binding of ICE1 to *CBF3* promoter to induce its expression and at the same time induces the degradation of ICE1 by HOS1 [28]. CBF3, in turn, activates GA 20-oxidases leading to a reduction of the gibberellin level and provokes the accumulation of DELLA proteins [76]. Consequently, DELLAs regulate jasmonate pathway signaling leading to the releasing of MYC2 transcriptional activity by interaction with JASMONATE ZIM-domain proteins [77]. Finally, MYC2, a key regulator of jasmonate-mediated responses, positively regulates the transcription of flavonoid biosynthetic genes and negatively regulates biosynthesis of indole GSLs [78]. Moreover, ICE1 physically interacts with ABSCISIC ACID INSENSITIVE5 (ABI5) to antagonize its transcriptional function [79]. ABI5 is the central regulator of abscisic acid (ABA) functions, participating in diverse abiotic stress responses, including biosynthesis of phytoalexins [80]. In particular, ABI5 regulates the transcriptional activity of *MYB28* and *MYB29*, pivotal transcriptional regulators of aliphatic GSLs biosynthesis [81]. ABI5 also participates in indolic GSLs metabolism by modulating the transcriptional activity of cytochrome P450 enzymes, CYP79B2 and CYP83B1, while having no effect on MYBs [82]. Moreover, ABI5 is implicated in the biosynthesis of flavonoids/anthocyanins as a positive regulator of *PAP1* and *PAP2* expression [83]. Similarly, in tomato, *Anthocyanins11*-mediated induction of secondary metabolites accumulation involves *ABI5* gene up-regulation [84]. It is noteworthy that DELLAs suppress the transcriptional function of ICE1 and its antagonistic effect on ABI5 to fine-tune ABA signaling [79]. Thus, several pathways can be affected either simultaneously or sequentially in *hos1* mutants. Besides, their interaction and intersection are also possible. In this regard, further research is needed to better understand the role of HOS1 in the regulatory relationships among light signaling, cold stress responses and of secondary metabolism.

## 4. Conclusions

In this report, Cas9-mediated genomic loss-of-function mutations of the *A. thaliana HOS1* genes were obtained for the first time. Several reading frame shifts occurred in the first exon resulting in premature termination of translation at codon positions 11, 23 and 24. Mutant plants, carrying *hos1* allele generated via CRISPR/Cas9 chemistry (*hos1*^Cas9^) and traditional T-DNA insertion mutagenesis (*hos1-3*) showed similar phenotypes, physiological and molecular responses to abiotic stress stimuli. Significant perturbations in phytoalexin biosynthesis have been detected, resulting in a reduced amount of GSLs and an elevated content of flavonoids. Moreover, *hos1* mutations provoked significant changes in expression of the corresponding bHLH and MYB transcription factors. To our knowledge this is the first evidence of HOS1 impact in the context of secondary metabolism. The results suggest the interplay between HOS1-mediated light signaling, responses to cold and the regulation of secondary metabolism in *Arabidopsis*.

## Figures and Tables

**Table 1:** Types and frequencies of identified Cas9-mediated mutations in *HOS1*.

Mutation Type	Predicted Protein Products of Mutant Alleles of the *HOS1* Gene Identified in This Work	Frequency among All Clones (%)	Mutant Plants
Insertion of C	MDTREINGFASAAEIDISSYAA#stop	31	*hos1-1* ^Cas9^
Insertion of A	MDTREINGFASADEIDISSYAA#stop	47	*hos1-2* ^Cas9^
Transition of C-to-T	MDTREINGFASAVRSISLPTQPNYSS	2.2	
Deletion of 30 bp	MDTR-------------------SISLPTQPNYSS	4.4	
Deletion of 35 bp	MDTREINGFA#stop	11	
Deletion of 71 bp	MDTREINGFASAARSISLPTQPN#stop	4.4	

**Table 2:** Accumulation of secondary metabolites (µmol/g of dry weight) in control and *hos1* mutants *A. thaliana* plantlets ^1^.

Secondary Metabolites	WT	*hos1-3*	*hos1-1* ^Cas9^	*hos1-2* ^Cas9^
**Flavonol glycosides**				
Quercetin hexose dideoxyhexose	0.11 ± 0.01 ^C^	0.21 ± 0.01 ^B^	0.91 ± 0.01 ^A^	2.45 ± 0.08 ^B^
Kaempferol hexose dideoxyhexose	1.18 ± 0.03 ^C^	1.17 ± 0.03 ^C^	5.16 ± 0.03 ^B^	8.32 ± 0.26 ^A^
Quercetin hexose deoxyhexose	0.75 ± 0.02 ^C^	1.37 ± 0.02 ^B^	1.66 ± 0.04 ^B^	3.90 ± 0.122 ^A^
Kaempferol hexose deoxyhexose	3.50 ± 0.29 ^C^	3.78 ± 0.28 ^C^	5.47 ± 0.42 ^B^	10.47 ± 1.32 ^A^
Isorhametin hexose deoxyhexose	0.56 ± 0.02 ^C^	1.72 ± 0.01 ^B^	1.99 ± 0.02 ^B^	4.96 ± 0.66 ^A^
Kaempferol dideoxyhexose	4.30 ± 0.53 ^C^	3.90 ± 0.55 ^C^	6.64 ± 0.82 ^B^	10.96 ± 1.35 ^A^
Quercetin deoxyhexose	0.31 ± 0.01 ^C^	1.60 ± 0.01 ^A^	0.41 ± 0.03 ^B^	0.43 ± 0.01 ^B^
Sum of flavonol glycosides	10.71	13.75	22.24	41.50
**Indolic glucosinolates**				
Indol-3-ylmethyl glucosinolate	10.74 ± 1.84 ^A^	10.36 ± 2.31 ^A^	9.15 ± 1.26 ^A^	11.67 ± 1.16 ^A^
4-Hydroxyindol-3-ylmethylglucosinolate	1.50 ± 0.06 ^A^	1.50 ± 0.04 ^A^	1.50 ± 0.05 ^A^	0.89 ± 0.02 ^B^
4-Methoxyindol-3-ylmethylglucosinolate	2.29 ± 0.07 ^A^	2.35 ± 0.07 ^A^	2.80 ± 0.06 ^A^	0.84 ± 0.02 ^B^
1-Methoxyindol-3-ylmethylglucosinolate	4.11 ± 0.69 ^A^	3.17 ± 0.64 ^A^	3.12 ± 0.13 ^A^	2.50 ± 0.16 ^B^
Sum of indolic glucosinolates	18.64	17.38	16.57	15.90
**Methyl-thioalkyl glucosinolates**				
4-Methylthiobutyl glucosinolate	14.80 ± 1.46 ^A^	7.65 ± 1.04 ^B^	7.07 ± 1.18 ^B^	6.68 ± 1.15 ^B^
5-Methylthiopentyl glucosinolate	1.24 ± 0.03 ^A^	0.43 ± 0.05 ^D^	0.74 ± 0.02 ^B^	0.60 ± 0.01 ^C^
7-Methylthioheptyl glucosinolate	1.27 ± 0.04 ^A^	0.52 ± 0.03 ^B^	0.41 ± 0.01 ^BC^	0.34 ± 0.01 ^C^
8-Methylthiooctyl glucosinolate	4.97 ± 0.54	0.87 ± 0.10	1.25 ± 0.03	0.84 ± 0.01
**Methyl-sulfinylalkyl glucosinolates**				
3-Methylsulfinylpropyl glucosinolate	9.76 ± 1.29 ^AB^	9.32 ± 1.21 ^AB^	7.93 ± 1.02 ^B^	12.77 ± 2.29 ^A^
4-Methylsulfinylbutyl glucosinolate	24.03 ± 2.69 ^AB^	21.28 ± 2.57 ^BC^	18.19 ± 1.49 ^C^	29.42 ± 2.69 ^A^
5-Methylsulfinylpentyl glucosinolate	2.41 ± 0.19 ^A^	1.96 ± 0.06 ^B^	1.78 ± 0.08 ^B^	3.42 ± 0.83 ^A^
6-Methylsulfinylhexyl glucosinolate	0.22 ± 0.01 ^A^	0.18 ± 0.01 ^B^	0.04 ± 0.01 ^C^	0.06 ± 0.01 ^C^
7-Methylsulfinylheptyl glucosinolate	1.20 ± 0.04 ^A^	0.71 ± 0.04 ^B^	0.22 ± 0.02 ^C^	0.25 ± 0.01 ^C^
8-Methylsulfinyloctyl glucosinolate	4.46 ± 0.84 ^A^	2.06 ± 0.17 ^B^	0.85 ± 0.07 ^C^	0.73 ± 0.02 ^C^
Sum of aliphatic glucosinolates	64.36	44.98	38.48	55.11
**Other glucosinolates**				
Pentyl glucosinolate	0.10 ± 0.01 ^C^	0.53 ± 0.03 ^A^	0.10 ± 0.01 ^C^	0.27 ± 0.02 ^B^
Phenethyl glucosinolate	0.11 ± 0.01 ^A^	0.07 ± 0.01 ^B^	0.07 ± 0.01 ^B^	0.14 ± 0.01 ^A^
Methylsulfonyloctyl glucosinolate	0.03 ± 0.01	tr	tr	tr
Hexyl glucosinolate	0.33 ± 0.02 ^A^	0.49 ± 0.05 ^A^	0.07 ± 0.01 ^B^	0.11 ± 0.01 ^B^
4-Benzoyloxybutyl glucosinolate	0.11 ± 0.01 ^B^	0.06 ± 0.01 ^C^	0.36 ± 0.01 ^A^	0.19 ± 0.02 ^B^
Total glucosinolates	83.70	63.53	55.63	71.70

^1^ Aerial part of 6-week-old control and transgenic plants from different passages were analyzed using tree biological replicates and two technical replicates. Data are presented as mean ± standard error. Different superscript letters indicate statistically significant differences of means (*P* < 0.05) in the columns, Fisher’s LSD. tr, trace amounts (below 0.01 µmol/g of dry weight).

## Data Availability

All data are contained within the article and supplementary materials.

## Supplementary Materials

The following are available online at https://www.mdpi.com/2223-7747/10/1/104/s1, Figure S1: In vitro target DNA cleavage using Cas9/sgRNA complex. Targ.—*AtHOS1* gene fragment was amplified with *HOS1-1*-target-F and *HOS1-2*-target-R primers and contained protospacer sequences for both gRNAs. gRNA1 and gRNA2 were obtained using HiScribe T7 Quick High-Yield RNA synthesis Kit (NEB, Ipswich, MA, USA), mixed with Cas9 nuclease (NEB) and *HOS1* DNA target. The mix was incubated at 37 °C for 15 min following fragment analysis using electrophoresis in 2% agarose gel, Figure S2: Visualization of GFP in *A. thaliana* seedlings subjected to *Agrobacterium*-mediated transformation using vacuum infiltration. Scanning by wavelengths (λ-scanning) to check the specificity of the fluorescence emission signal, Figure S3: Stability analysis of the candidate reference genes in *A*. *thaliana* calculated by RefFinder. Values above the bars indicate geomean of ranking values, Figure S4: Schematic illustration of the T-DNA insertion in *hos1-3* mutant line of *Arabidopsis thaliana* (SALK_069312C) purchased from the collection of the Salk Institute. The detail underneath shows partial sequence of the mutated *HOS1* allele obtained from the DNA template of *hos1-3* plant using PCR with LP and LBb primer pair. Intronic region is marked with blue color, exon is yellow-colored and T-DNA region is highlighted in grey. The 29-bp insertion of unknown origin was also found between the exon and the T-DNA sequences. T-DNA left border (LB) as well as LP and LBb primers binding sites are underlined. The predicted sequence of the truncated protein product is also shown below. The portion of the native HOS1 is italicized, Table S1: List of primer sequences used in this study.
