# Supplementary material for: CRISPR/Cas9-Mediated Knockout of HOS1 Reveals Its Role in the Regulation of Secondary Metabolism in Arabidopsis thaliana
Source: Plants (Basel). 2021 Jan 6;10(1):104. doi: 10.3390/plants10010104 (PMC7825447; doi:10.3390/plants10010104)
Supplement: Supplementary file 1 [file plants-10-00104-s001.zip › Supplementary Materials.docx]

**Supplementary Materials**


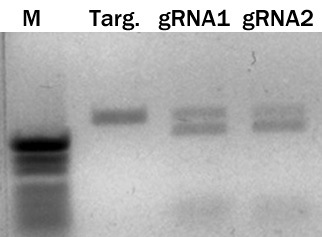


**Figure S1.** *In vitro* target DNA cleavage using Cas9/sgRNA complex. Targ. - *AtHOS1* gene fragment was amplified with *HOS1-1*-target-F and *HOS1-2*-target-R primers and contained protospacer sequences for both gRNAs. gRNA1 and gRNA2 were obtained using HiScribe T7 Quick High-Yield RNA synthesis Kit (NEB, Ipswich, MA, USA), mixed with Cas9 nuclease (NEB) and *HOS1* DNA target. The mix was incubated at 37^o^C for 15 min following fragment analysis using electrophoresis in 2% agarose gel.


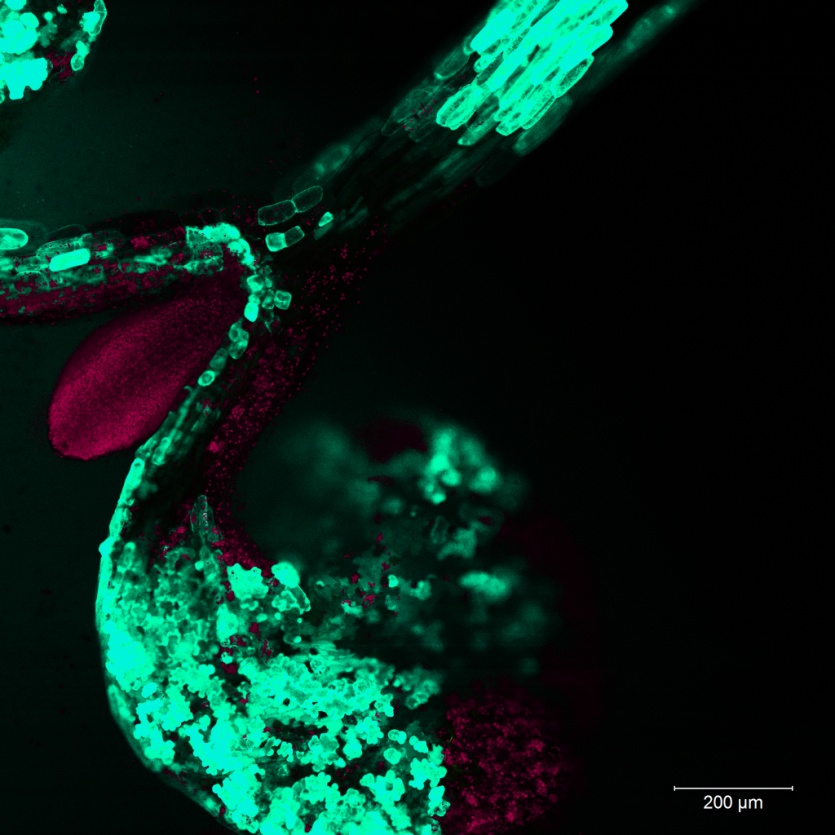


**Figure S2.** Visualization of GFP in *A. thaliana* seedlings subjected to *Agrobacterium*-mediated transformation using vacuum infiltration. Scanning by wavelengths (λ-scanning) to check the specificity of the fluorescence emission signal.


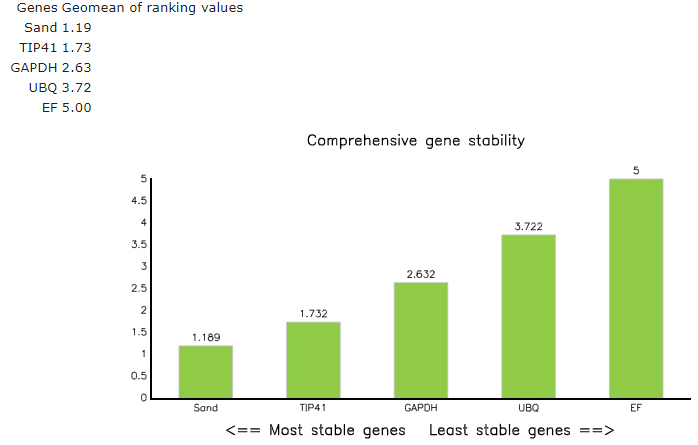


**Figure S3.** Stability analysis of the candidate reference genes in *A*. *thaliana* calculated by RefFinder. Values above the bars indicate geomean of ranking values.


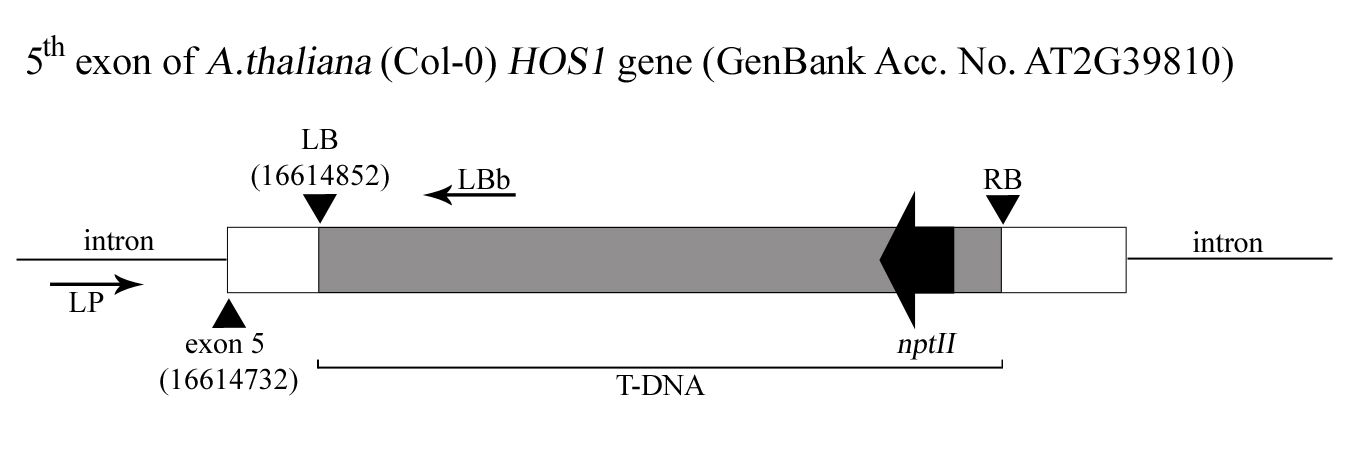


**LP>**AAGGCACGATAATGGTCTTCCATCATCAGCTTGGATCTGATTTTCTATCCCTAGCTTTGTGACTTTATTCTTCTTTTCTTTTTTAATGTCATTGCATATTAGTGAATGTAGATGGGGTATATGTAACCTATCTTTATATGACATACTGAATAGTAAATGAGTATAACCTTATGGCTTCTGACCAGAGAGTGTTTTAAATAGATCAGCTCTTTAGCTATTGGTGCAAGATCACTGAGCTTACGTGTGCTTACCCTATTCTTTTTATTTTATTTCAGCATTTGGACATAATGGTCTGGTGCATCAGACATGGATTTCTGGATGATGTGAGGTCTCGATATTCGAATTTCACATCGTGGAATGCACTGGTAGGGGAAAGAAAATCAAATGCAGTTAAGCAAATCTTGCGGACAAATTGTCTAAGCTGTTGCCAGGATATATTGTGGTGTAAACAAATTGACGCTTAGACAACTTAATAACACATTGCGGACGTTTTTAATGTACTGGGGTGGTTTTTCTTTTCACCAGTGAGACGGGCAACAGCTGATTGCCCTTCACCGCCTGGCCCTGAGAGAGTTGCAGCAAGCGGTCCACGCTGGTTTGCCCCAGCAGGCGAAAATCCTGTTTGATGGTGGTTCCGAAATCGGCAAAAT**<LBb**

*MDTREINGFASAARSISLPTQPNYSSKPVQEALKHLASINLRELCNEAKVERCRATRDLASCGRFVNYVLNPCGHASLCTECCQRCDVCPICRSTLPKFGDRLRLRLYYECVEAGLISRTHEEASQDSDEDEHQLAADVHRLYSLFDVAMNNNLISVVCHYITNVCMDETAVSSDPVIAFLLDEVVVKDWVKRTFRSTLAELQEIYNLETKEMQAWLDKLLRCSKQVAGICSVLEVMESAFKGSVSPQLQDVQTLRENIGKTKQHLDIMVWCIRHGFLDDVRSRYSNFTSWNALVGERKSNAVK*QILRTNCLSCCQDILWCKQIDA#stop

**Figure S4**. Schematic illustration of the T-DNA insertion in *hos1-3* mutant line of *Arabidopsis thaliana* (SALK_069312C) purchased from the collection of the Salk Institute. The detail underneath shows partial sequence of the mutated *HOS1* allele obtained from the DNA template of *hos1-3* plant using PCR with LP and LBb primer pair. Intronic region is marked with blue color, exon is yellow-colored, and T-DNA region is highlighted in grey. The 29-bp insertion of unknown origin was also found between the exon and the T-DNA sequences. T-DNA left border (LB) as well as LP and LBb primers binding sites are underlined. The predicted sequence of the truncated protein product is also shown below. The portion of the native HOS1 is italicized.

**Table S1**. List of primer sequences used in this study.

| Primer name  (GenBank accession no.) | Forward (5′ to 3′) | Reverse (5′ to 3′) |
| --- | --- | --- |
| Construction of sgRNA expression cassettes | | |
| gRNA1 | GAAGAGATATCGATCTCGCGGGTTTTAGAGCTAGAAATAGC | CCGCGAGATCGATATCTCTTCAATCACTACTTCGTCTCTAAC |
| gRNA2 | GGTAGCGTACTTCTACAGATGTTTTAGAGCTAGAAATAGC | ATCTGTAGAAGTACGCTACCAATCACTACTTCGTCTCTAAC |
| pUC119-CeuI | CATTCGCTACCTTAGGACCGTTATAGTTAGAGCTCAGAAATCTCAAAATTC | ATGTAACTATAACGGTCCTAAGGTAGCGAGTACAAGAAAGCTGGGTCTAG |
| PCR templates for *in vitro* transcription | | |
| T7-gRNA1 | TAATACGACTCACTATAGGGAAGAGATATCGATCTCGCGG | GCACCGACTCGGTGCCAC |
| T7-gRNA2 | TAATACGACTCACTATAGGGGGTAGCGTACTTCTACAGAT | GCACCGACTCGGTGCCAC |
| Mutant screening using HMA, HRM and sequencing | | |
| *HOS1-1-*target  (AT2G39810, 1st exon) | CAGTGTTTCCTCTCTTCGTC | TAGATGCCAAATGTTTCAACG |
| *HOS1-2-*target  (AT2G39810, 2nd exon) | ACAGTAGCAAGCCTGTTCAG | GTCTATCTCCAAATTTTGGT |
| qPCR analysis | | |
| *FLC* (AT5G10140) | CTGCCCTCTCCGTGACTAG | GGAGAGTCACCGGAAGATTG |
| *FLT* (AT1G65480) | CCACTGCAGGAATTCATCGT | AGTCTTCTTCCTCCGCAGC |
| *TSF* (AT4G20370) | ACTGGTTGGTGACTGATATAC | GCAGTTGAAGTAAGAGGCAG |
| *COP1* (AT2G32950) | GGAAGCGAGACAAACGAAGT | CACGCAGCGAGTACCAGAAC |
| *CO* (AT5G15840) | GACTCACTACAACGACAATG | CTTGTCTTCCTCTTCTCTCTG |
| *DREB1B*  (*CBF1*) (AT4G25490) | GTCAACATGCGCCAAGGATA | TCGGCATCCCAAACATTGTC |
| *DREB1C*  (*CBF2*) (AT4G25470) | GAATCCCGGAATCAACCTGT | CCCAACATCGCCTCTTCATC |
| *DREB1A*  (*CBF3*) (AT4G25480) | ACTTGCGCTAAGGACATCCA | TGTCGAAGCCATGATCCGTC |
| *COR6.6*  (*KIN2*) (AT5G15970) | GACCAACAAGAATGCCTTCC | TGCTCTTCTCCTCAGCTTTGC |
| *COR15a* (AT2G42540) | AACGAGGCCACAAAGAAAGC | GCTTCTTTACCCAATGTATCTG |
| *COR15b* (AT2G42530) | AACGAAGCCACAAAGAAAGC | CCATCCGCCAAGGCCTCC |
| *COR47* (AT1G20440) | AGCCTAGTGTCATCGAAAAG | CTTCATCGCTCGAAGAGGAAG |
| *COR78*  (*RD29A*) (AT5G52310) | ACCAGGCGTAACAGGTAAAC | AAACACCTTTGTCCCTGGTGG |
| *ICE1* (AT3G26744) | AGGAAAGGGTAAGAAGAAAG | GGACAACTGATCTAAGCATAT |
| *MYB11* (AF062863) | CCAACTGTCGCCAATACCGTCG | CAATCGCCATCGTCTCCATAAC |
| *MYB12* (NM_130314) | GTGCGGTGGTGATGGGGAG | CTTGGTGGTCGTAGTTGAG |
| *PAP1* (NM_104541) | CTCAATGCCCCACCAAAAG | CCCCCTTTTCTGTTGTCGTC |
| *TT8* (NM_117050) | GTAAGAGAAAAACATCGGAGG | GTGAGGCTAAAACAAAAATACG |
| *TTG1* (NM_180738) | GTTCTGGTGGTGATGATAC | CTAAAAGCGAGAGGGAGCG |
| *MYB34* (*ATR*) (At5g60890) | TGGTGAAGGTGGATGGCGT | GATAGTGTCGTCTTCTTCAGG |
| *MYB51* (AAF98417) | GGAAAGAGAATCAATCAGAGTG | GAAATGTGGAGAACCCGATAG |
| *MYB122* (NM_10606) | GTTCTTCCTCCACACATACC | ATGTCACCGACTATTTCCTC |
| *MYB28* (NM_125535) | CCTTCTCCCCATCATCGTTG | CATTCATACTGTTCTCCTCATTG |
| *MYB76* (NM_120852) | GATGACGACGAAAATGAGAAC | GGGATGGTCAAGAAGATAAG |
| *UBQ* (AT4G05320) | GGCCTTGTATAATCCCTGATG | GAGATAACAGGAACGGAAAC |
| *GAPDH* (AT1G13440*)* | TTGGTGACAACAGGTCAAGC | AACTTGTCGCTCAATGCAATC |
| *EF-1α* (AT5G60390) | GAGCACGCTCTTCTTGCTTTC | TGGTGGCATCCATCTTGTTAC |
| *TIP41* (AT4G34270) | GAAAACTGTTGGAGAGAAGC | TCAACTGGATACCCTTTCGCA |
| *MON1*  (*Sand*)  (AT2G28390) | CTCTATGCAGCATTTGATCCA | ATTGCATATCTTTATCGCCATC |
